# A Study on the Correlations between Musculoskeletal Disorders and Work-Related Psychosocial Factors among Nursing Aides in Long-Term Care Facilities

**DOI:** 10.3390/ijerph19010255

**Published:** 2021-12-27

**Authors:** Man-Hua Yang, Chao-Jie Jhan, Pei-Chi Hsieh, Chieh-Chun Kao

**Affiliations:** 1 Institute of Clinical Nursing, College of Nursing, National Yang Ming Chiao Tung University, Taipei 11221, Taiwan; mhyang@nycu.edu.tw; 2 Institute of Clinical Nursing, School of Nursing, National Yang Ming University, Taipei 11221, Taiwan; a17480456@gmail.com; 3 Department of Nursing, Hungkuang University, Taichung 43304, Taiwan; peichihsieh2018@gmail.com; 4 Department of Nursing, Ching Kuo Institute of Management and Health, Keelung 20301, Taiwan

**Keywords:** musculoskeletal disorders, psychosocial factors, nursing aides, job content questionnaire, assistive device

## Abstract

Background: Among the nursing aides employed at long-term care facilities (LTCFs), those with musculoskeletal disorders (MSDs) are most likely to experience disability or develop an intention to leave. The purpose of this study was to investigate the association of work-related psychological factors among nursing aides in LTCFs with MSDs in Taiwan. Methods: Purposive sampling was used in this cross-sectional study to enrol 308 nursing aides from residential LTCFs in Taiwan as research subjects. A demographic and job background survey, a job content questionnaire (JCQ), and the Nordic musculoskeletal questionnaire (NMQ) were used to collect data. Results: Lower job control associated with higher psychological job demands, and lower social support was associated with more severe MSDs for the nursing aides (*p* < 0.001). Among the MSDs reported by nursing aides in LTCFs, lower back pain was the most serious. In addition, nationality, age, exercise habits, chronic diseases, worksite, lack of rest time, lack of assistive devices, low coworker support, and high psychological job demands were significant factors affecting MSDs. In total, 42.1% of the variance in MSDs among nursing aides in LTCFs was explained. Conclusions: Work-related psychological factors among nursing aides in LTCFs have an important association with MSDs. For nursing aides, coworker support should be improved, and their psychological demands at work should be reduced.

## 1. Introduction

Taiwan is an Asian country with an elderly population that totals 14.1% of the national population. The long-term shortage of nursing aides makes caregivers a high-risk group with high psychosocial stress and a high incidence of musculoskeletal disorders (MSDs) [1]. More than 88% of nursing aides in long-term care facilities (LTCFs) suffer from MSDs, and the prevalence of MSDs among nursing aides in general has been reported to be as high as 76.9% to 92.3% [2]. MSDs increase the risk of chronic diseases, lead to a reduction in working hours and early retirement, and lead to disability among nursing aides, resulting in a heavy burden on the medical care system and society [1,3].

Many factors may influence the development of MSDs, including advanced age, female sex, high body mass index (BMI), lack of exercise, smoking habits, and chronic diseases [4,5]. With their heavy workloads and extensive walking requirements, nursing aides are prone to lower limb MSDs. The severity of upper limb MSDs is increased among nursing aides in positions that require them to assist patients in taking baths or turning over or to lift heavy objects at work [5,6]. The most common areas of MSD in nursing aides are the shoulders, neck, and lower back, and the prevalence of soreness increases significantly with age, seniority, and education. Lower back pain tends to occur in caregivers 25–34 years of age who use few assistive devices and lack exercise habits [5,7].

MSDs may also be related to psychosocial factors [8,9,10]. The job demands-control model developed by Karasek et al. [11] aims to understand the pressure and work-related psychological factors among workers based on job control, job demands, and social support. Surveys have shown that people with low job control or high job demands are prone to higher MSDs and lower job satisfaction [12,13]. Without social support, workers with social isolation experience higher job demands and poor physical functioning [11].

Currently, studies investigating the association of work-related psychological factors with MSDs in nursing aides are lacking. The purpose of this study was to investigate the association of demographic characteristics, job background, and work-related psychological factors with MSDs among nursing aides in LTCFs.

## 2. Materials and Methods

### 2.1. Study Design, Sample, and Setting

The present study was a cross-sectional survey. From 5 March to 30 July 2020, simple random sampling was used to select 15 residential LTCFs in Taiwan from the list of LTCFs released by the Ministry of Health and Welfare, and purposive sampling was used to enrol subjects from the selected study sites. The inclusion criteria for nursing aides were as follows: employment at an LTC facility for at least 1 year, at least a junior high school graduate educational background, and a Nordic musculoskeletal questionnaire (NMQ) score of at least two points for one area. The exclusion criteria for nursing aides were as follows: history of MSDs and member of the administrative staff.

Regarding ethical considerations, the researcher personally explained the research purpose and data collection method to the research subjects and obtained consent before initiating data collection. For data collection, after completing the questionnaire anonymously, each research subject sealed it in the provided envelope and sent it back to the researcher.

The sample size needed for this study was calculated using G*Power statistical power analysis software. The sample size calculation was based on a *t* test-correlation and the point biserial model. The parameters were set to α = 0.05, effect size = 0.15, and a statistical power level = 0.8. The results indicated that a minimum of 270 original samples were required. After taking into account an estimated 10% sample loss rate, the effective sample size was set at 297.

### 2.2. Measurements

#### 2.2.1. Demographic Characteristics and Job Background Survey

This survey gathered information about sex, nationality, age, BMI, exercise habits, chronic disease, workplace, worksite, seniority, working hours, job pattern (i.e., fixed work schedule, regular shifts), rest time, use of protective aids, and use of lifting aids.

#### 2.2.2. Job Content Questionnaire (JCQ)

The Chinese version of the JCQ was used and includes 3 dimensions: job control, psychological job demands, and social support. Job control includes 9 items in total, which are associated with skill discretion (SD) and decision authority (DA). Psychological job demands include 7 items. Social support includes 8 items in total, which are associated with supervisor support and coworker support. Some of the items are reverse items, meaning that the scores are reversed before statistical analysis. With the exception of the “supervisor and coworker support” subscale, which was calculated using the original scoring method, the scores of the remaining subscales were obtained using the weighted scores of the respective items. A higher total score of a dimension indicated greater job control, higher job demands, and higher social support. Cronbach’s alpha of the internal consistency of the questionnaire was 0.69–0.86, and Pearson’s correlation coefficients of test–retest reliability were 0.62–0.72 (*p* < 0.01) [14].

#### 2.2.3. NMQ

The Chinese version of the NMQ was used to measure the subjects’ intensity, duration, and frequency of pain in 11 musculoskeletal areas, where “0” denoted no pain and “5” denoted severe pain; severe pain was defined as being unable to move or pain lasting for more than 14 days during the past six months. In this study, the intensity of pain was used as an indicator for statistical analysis. The reliability intraclass correlation coefficient (ICC) of the questionnaire was 0.97, the Kappa value was 0.71–0.96, the reliability of the Chinese version of the questionnaire was between 0.77 and 1.0, and the Cronbach’s alpha was between 0.8 and 1.0 [15,16]. Moreover, based on the characteristics of MSDs and a literature review [5,17], the 11 areas were categorized into 4 categories, namely, the shoulder, neck and upper back area; the upper limb area (hands, forearms); the lower back area; and the lower limb area [17]. The formula (score of each category/total score)∗100% was used to calculate the score percentage for each area.

### 2.3. Statistical Analyses

SPSS for Windows version 22.0 (IBM Corp., Armonk, NY, USA) was used for data analysis, including the calculation of descriptive statistics, independent t test, one-way ANOVAs, and the calculation of Pearson’s correlation coefficients. Furthermore, hierarchical regression analysis was conducted to identify the amount of variation in MSDs among nursing aides in LTCFs. In the hierarchical regression analysis, the dependent variable was the MSD score, and the independent variables were demographic characteristics and the job background of nursing aides, which have reached significance variables, and the work-related psychological factors. The Kolmogorov–Smirnov normality test, Quantile–Quantile plot, Durbin–Watson test, and Residual Plot test were used to check whether the residual errors of regression were normally distributed, independent and homoscedastic. According to the collinearity diagnostics, tolerance results > 0.1 and variance inflation factor (VIF) results < 10, no collinearity was associated with the three models used in this study.

## 3. Results

### 3.1. Analysis of the Demographic Characteristics, Job Background, and MSDs among Nursing Aides in LTCFs

As shown in Table 1, 308 nursing aides participated in this study, of whom 89.6% were female. The mean age was 44 (SD = 13.6). Most of the nursing aides had normal BMI, did not have exercise habits, and suffered from at least one chronic disease. The most common chronic diseases of the subjects were hypertension, heart disease, and diabetes. Seventy-five percent of the research subjects worked in a nursing home (*n* = 231), forty-nine percent of the research subjects worked in the middle of Taiwan (*n* = 151), the mean seniority was 6.5 years (SD = 5.9), and the mean number of working hours was 10.3 hours per day (SD = 3.2). Among the nursing aides in LTCFs, 39.6% job pattern had regular shifts, 18.2% had no fixed rest time during their shifts, 40.2% did not use protective assistive devices, and 61.4% never used lifting aids. The NMQ scores of the research subjects were highest for the lower back area and lowest for the lower limb area.

### 3.2. Correlations between Work-Related Psychological Factors and MSDs in the Four Areas among Nursing Aides in LTCFs

As shown in Table 2, job control was significantly negatively correlated with MSDs in all four areas (*p* < 0.001). Psychological job demands were significantly positively correlated with MSDs in all four areas (*p* < 0.001). Social support was significantly negatively correlated with MSDs in all four areas (*p* < 0.001). The results showed that lower job control was associated with higher psychological demands of the job and that lower social support was associated with more severe MSDs of the nursing aides (*p* < 0.001).

### 3.3. Associations of Demographic Characteristics and Job Background with MSDs in the Four Areas among Nursing Aides in LTCFs

The results showed that the NMQ scores for all four areas were significantly higher in the research subjects with chronic disease than in those without chronic disease. Vietnamese, advanced age, and working in nursing homes had significantly higher NMQ scores in the lower back area than Indonesian, 50–64 years old, and not working in nursing homes. For those working in northern Taiwan, the upper limb area had significantly higher NMQ scores than those working in middle Taiwan. Nursing aides without exercise habits had significantly higher NMQ scores in the shoulder, neck, and upper back area than those who exercised at least once a week. In addition, the research subjects who had no rest time at work had significantly higher NMQ scores for the shoulder, neck, and upper back area, the upper limb area, and the lower limb area. Compared with nonshift workers, shift workers had significantly higher NMQ scores for the shoulder, neck, and upper back area and the upper limb area. Research subjects who did not use protective assistive devices or lifting assistive devices had significantly higher NMQ scores for the upper and lower limb areas than those who used such devices. The NMQ scores for sex, BMI, seniority, and working hours in the four areas did not reach statistically significant differences. The results showed that nursing aides in LTCFs who were Vietnamese, aged over 65, lacked of exercise habits, had chronic disease, working in nursing homes, working in northern Taiwan, lacked rest at work, long-term shift work, and no use of assistive devices had a higher chance of suffering from MSDs, as shown in Table 3.

### 3.4. Prediction and Analysis of Total MSDs among Nursing Aides in LTCFs

This study further put the demographic characteristics and the job background of nursing aides which have reached significance variables, and the work-related psychological factors into a regression model for hierarchical regression analysis of the total MSDs among nursing aides in LTCFs (as shown in Table 4). 

The results of model l (which included demographic characteristics as explanatory variables) showed that the presence of nationality, age, chronic disease, and worksite significantly affected MSDs of nursing aides (F = 8.58, *p* < *0*.001), with an explanatory power of 26.0%. The results of model 2 (which included the model 1 variables and job background variables) showed that rest time, protective aids, and lifting aids significantly affected MSDs of nursing aides (F = 5.04, *p* < *0*.001), and the variance explained was 35.2%.The results of model 3 (which included the model 2 variables and work-related psychological factors) showed that there was a significant change in R^2^ (F = 6.73, *p* < 0.001) from model 2, and the explanatory power for MSDs of nursing aides increased to 42.1%. Coworker support had significantly negative predictive power for MSDs of nursing aides, and psychological job demands had significantly positive predictive power for MSDs of nursing aides.

## 4. Discussion

In this study, the results of the hierarchical regression analysis revealed that work-related psychological factors among nursing aides were associated with MSDs. The lower the social support of nursing aides and the higher their psychological job demands, the higher the severity of MSDs. This result is similar to previous studies exploring the correlation between psychological factors and MSDs in nursing aides, which found that high psychological stress, low social support, and effort–reward imbalance were significantly associated with MSDs [2]. This result is consistent with the job demands–control model. A health survey in the U.S. discovered that the psychological health of workers with a low-strain job pattern is better than that of workers with a high-strain job pattern [11]. In addition, the research results showed that the severity of MSDs in the lower back area was the highest among nursing aides. In another study, the chance of lower back pain was found to be higher in a high load-low control group than in a low load-high control group [2]. The above results are all consistent with the results in the present study. Abdullah et al. [18] investigated the factors causing lower back pain in the occupational environment of caregivers and found that perceived general tension is an important variable in both directly influencing lower back pain and indirectly influencing lower back pain through other stress-related variables, which suggests that this work-related psychological factor is an important factor affecting MSDs.

In addition to revealing the influences of sociopsychological factors, the results of this study revealed that older nursing aides had the highest NMQ scores in the lower back area and that older nursing aides had higher levels of MSDs than younger nursing aides. Previous studies have found that advanced age is a significant predictor of musculoskeletal disorder [1], and this study showed similar results. The mean age of the research subjects was 44.0 ± 13.6 years old, with only 28.6% under the age of 35. According to the 2020 Statistics Report of the Executive Yuan, Taiwan the average age of workers in Taiwan is 40.3 years old, and nursing aides are classified as an ageing job category, with an ageing trend. Therefore, MSDs and other health problems in older nursing aides should be monitored and tracked on an ongoing basis.

This study found that the NMQ scores in the four areas of subjects with chronic disease as a demographic characteristic were significantly higher than those of subjects without chronic disease (*p* < 0.001). The presence of two chronic diseases was significant factor affecting MSDs. A possible explanation for this result is that insufficient activity level and obesity affect the immune system and inflammatory reactions, which result in chronic diseases and musculoskeletal diseases [19]. Salter et al. [20] found that MSDs can reduce patient activity levels and increase the risk of cardiovascular disease and diabetes. Many other studies have also verified that, in addition to causing reduced activity, obesity, and chronic inflammation, chronic diseases are likely to cause cardiovascular and musculoskeletal diseases [4,19]. These results are all consistent with those of the present study [21].

On the other hand, the results of this study found that the NMQ scores in the upper limb area were significantly higher for nursing aides working in the northern of Taiwan than for those working in the middle of Taiwan. This trend was not found in previous studies, so a cross-tabulation of relevant factors revealed that nursing aides in northern Taiwan had more chronic disease (30.1%) than those in middle Taiwan (20.5%). After using Pearson’s correlation coefficient, a significant difference (*p* = 0.031) was reached, which may be one of the reasons for the significantly higher NMQ scores of the northern nursing aides compared to those in the middle region. In addition, previous studies found that exercise can improve indicators of metabolic syndrome such as BMI, high-density cholesterol, and triglycerides, as well as increase muscle strength and endurance, which can help to reduce the incidence of MSDs [22,23]. The results of the hierarchical regression analysis also revealed that nursing aides who exercised at least three times a week had significantly fewer MSDs than those who did not exercise.

This study revealed that some of the job background characteristics of nursing aides, such as a lack of rest time, long-term shift work, and little or no use of aids, were more likely to be associated with MSDs. This result is consistent with those of previous studies [7,24,25]. This study also revealed that lifting aids significantly reduced the occurrence of MSDs of the shoulder, neck, and upper back area; upper limb area, and lower limb area, while protective aids significantly reduced the occurrence of MSDs of the upper and lower limbs. These results may be related to the results of previous studies, which reported that the use of protective gear can provide protection, reduce joint stress, and maintain muscle stability. The use of shower trolleys, overhead lifts, and nonslip mats by nursing aids when performing care work may help reduce work intensity and the frequency at which they need to push or pull the patient, thereby reducing MSDs [26,27].

### Limitations and Suggestions

This study adopted a cross-sectional research method and did not perform any follow-up on the long-term effects of demographic characteristics and job background on MSDs among nursing aides in LTCFs.

Moreover, because the research subjects were nursing aides in LTCFs, the conclusions drawn from the research results are limited to this type of workplace. In addition, comprehensive data on disease severity and physical activity functions among residents receiving care from nursing aides could not be collected, in part because some of the supervisors were unwilling to help with enrolment for this study. The absence of these data might have affected the research results. On the other hand, we used scales to evaluate MSDs, and a limitation may be the lack of objective measures.

Approximately 40% of the nursing aides enrolled in this study were foreign workers. Due to language barriers, this study only used a structured questionnaire for the survey and did not assess the association of differences in nationality or culture with work-related psychological factors. Future studies should analyse how cultural differences between Taiwanese and foreign nursing aides affect the occurrence of MSDs.

## 5. Conclusions

The research results show that work-related psychological factors among nursing aides in LTCFs have an important association with MSDs. In particular, the association of coworker support and psychological job demands were the most significant. The MSDs of nursing aides in LTCFs with low job control, high psychological job demands, and low social support were more serious than those of nursing aides without these characteristics. In consideration of the research results, managers of LTCFs are advised to strengthen preoccupational training and organizational decision-making participation among nursing aides according to the job items and to set reasonable working hours to enable nursing aides to obtain sufficient rest. In addition, managers of LTCFs should actively provide caregivers with protective aids, such as waist supports, knee supports, and lifting aids such as overhead lifts, shower trolleys, and handling belts. Furthermore, managers of LTCFs should increase communication channels and support networks to alleviate MSDs in nursing aides.

## Figures and Tables

**Table 1:** Analysis of the demographic characteristics, job background, and MSDs among nursing aides in LTCFs (*n* = 308).

Variable	Range	M ± SD	*n* (%)
Sex			
Female			276 (89.6)
Male			32 (10.4)
Nationality			
Taiwanese			195 (63.3)
Vietnamese			78 (25.3)
Indonesian			35 (11.4)
Age (years)	19–77	44.0 ± 13.6	
20–34			88 (28.6)
35–49			106 (34.4)
50–64			89 (28.9)
65 and above			25 (8.1)
BMI			
BMI < 18.5			10 (3.2)
18.5 ≤ BMI < 24			173 (56.2)
24 ≤ BMI			125 (40.6)
Exercise habit			
None			196 (63.6)
≥1 time/week			71 (23.1)
≥3 time/week			41 (13.3)
Chronic disease			
None			222 (72.1)
1 type			55 (17.9)
≥2 types			31 (10.1)
Workplace			
Care facility			31 (10.1)
Nursing facility			46 (14.9)
Nursing home			231 (75.0)
Worksite			
Northern			97 (31.5)
Middle			151 (49.0)
Southern			60 (19.5)
Seniority (years)	1–30	6.5 ± 5.9	
Working hours (hours/day)	6–24	10.3 ± 3.2	
Job pattern			
Fixed work schedule			186 (60.4)
Regular shifts			122 (39.6)
Rest time			
No			56 (18.2)
Yes			252 (81.8)
Use of protective aids			
No			124 (40.2)
Yes			184 (59.7)
Use of lifting aids			
No			189 (61.4)
Yes			119 (38.6)
MSD area		M ± SD	
Shoulder, neck, and upper back		24.8 ± 14.1	
Upper limb		26.9 ± 14.1	
Lower back		41.7 ± 18.4	
Lower limb		18.9 ± 14.9	

**Table 2:** Results of the correlation analyses of work-related psychosocial factors and the four MSD areas among nursing aides in LTCFs (*n* = 308).

Variable	Mean	SD	Shoulder, Neck, and Upper Back Area		Upper Limb Area		Lower Back Area		Lower Limb Area	
			*r*	*p*	*r*	*p*	*r*	*p*	*r*	*p*
Skill discretion	30.9	4.9	−0.45	<0.001	−0.39	<0.001	−0.33	<0.001	−0.40	<0.001
Decision authority	29.3	6.2	−0.37	<0.001	−0.40	<0.001	−0.40	<0.001	−0.38	<0.001
Psychological job demands	32.0	3.9	0.48	<0.001	0.44	<0.001	0.44	<0.001	0.51	<0.001
Supervisor support	11.4	2.2	−0.40	<0.001	−0.28	<0.001	−0.34	<0.001	−0.38	<0.001
Coworker support	11.8	2.1	−0.40	<0.001	−0.34	<0.001	−0.28	<0.001	−0.39	<0.001

Note: *r*: Pearson’s correlation coefficient.

**Table 3:** Association of demographic characteristics and job background with MSDs in the four areas among nursing aides in LTCFs (*n* = 308).

Variable	Shoulder, Neck, and Upper Back Area			Upper Limb Area			Lower Back Area			Lower Limb Area		
	M ± SD (%)	*F/t*	*p*	M ± SD (%)	*F/t*	*p*	M ± SD (%)	*F/t*	*p*	M ± SD (%)	*F/t*	*p*
Nationality		1.91	0.127		2.34	0.075		4.41	0.005 **		2.60	0.053
① Taiwanese	25.7 ± 12.3			28.0 ± 14.2			42.0 ± 18.5		② > ③	20.4 ± 15.5		
② Vietnamese	25.3 ± 16.5			26.7 ± 14.9			46.0 ± 18.4			18.4 ± 15.6		
③ Indonesian	19.3 ± 12.8			23.1 ± 10.0			30.0 ± 12.7			12.6 ± 7.2		
Age (years)		1.94	0.124		2.18	0.092		3.98	0.009 **		1.24	0.280
① 20–34	23.7 ± 15.9			26.7 ± 13.1			39.2 ± 17.5		④ > ③	17.6 ± 14.0		
② 35–49	25.6 ± 15.1			26.2 ± 14.3			44.2 ± 19.7			18.3 ± 14.7		
③ 50–64	23.1 ± 10			25.2 ± 13.4			37.9 ± 14.1			18.3 ± 14.7		
④ ≥65	30.9 ± 15.9			34.7 ± 16.3			52.7 ± 24.1			21.5 ± 20.6		
Exercise Habit		4.13	0.017 *		0.28	0.75		2.43	0.090		2.19	0.114
① None	26.5 ± 14.8		① > ②	27.4 ± 13.9			43.2 ± 19.5			20.2 ± 15.7		
② ≥1 time/week	20.5 ± 12.4			25.3 ± 12.8			35.8 ± 15.1			15.0 ± 12.1		
③ ≥3 time/week	23.4 ± 11.6			17.8 ± 4.0			41.3 ± 15.3			17.1 ± 13.4		
Chronic diseases		18.20	<0.001 ***		13.85	<0.001 ***		10.34	<0.001 ***		32.04	<0.001 ***
① None	21.8 ± 12.2		③ > ②	23.4 ± 11.0		③ > ②	37.8 ± 16.4		③ > ②	14.0 ± 10.5		
② 1 type	28.1 ± 14.0		② > ①	29.7 ± 16.2			45.3 ± 19.1		② > ①	22.7 ± 15.7		
③ ≥2 types	37.2 ± 16.5			37.9 ± 16.7			53.7 ± 20.4			34.3 ± 18.1		
Workplace		0.17	0.848		0.42	0.565		6.84	<0.001 ***		0.59	0.555
① Care facility	25.6 ± 14.04			24.3 ± 16.3			31.8 ± 11.3		③ > ②	15.8 ± 14.9		
② Nursing facility	23.6 ± 11.3			26.3 ± 10.9			35.4 ± 15.5		② > ①	19.1 ± 14.0		
③ Nursing home	24.8 ± 14.6			27.4 ± 14.4			44.3 ± 19.1			18.8 ± 14.9		
Worksite		1.24	0.291		6.18	0.003 **		0.93	0.390		0.47	0.626
① Northern	26.6 ± 14.4			31.9 ± 18.0		① > ②	41.9 ± 21.4			20.3 ± 17.4		
② Middle	24.4 ± 13.1			23.6 ± 10.2			40.2 ± 15.1			18.3 ± 13.0		
③ Southern	23.0 ± 15.7			26.5 ± 12.9			45.1 ± 20.5			18.3 ± 15.0		
Job pattern		−2.07	0.022 *		−2.38	0.018 *		−1.67	0.065		−1.47	0.099
① Fixed working schedule	23.2 ± 13.1			24.8 ± 13.1			39.9 ± 17.4			17.5 ± 13.6		
② Regular shifts	27.1 ± 15.1			30.2 ± 15.1			44.2 ± 19.6			20.6 ± 16.4		
Rest time		−4.13	0.007 **		−2.74	<0.001 ***		−4.31	0.089		−2.82	0.001 **
① No	22.7 ± 12.4			25.0 ± 12.6			39.1 ± 16.3			16.9 ± 12.9		
② Yes	33.5 ± 17.1			32.5 ± 16.7			52.5 ± 22.5			25.4 ± 19.1		
Use of protective aids		2.75	0.092		1.79	0.028 *		2.11	0.067		2.86	<0.001 ***
① No	27.6 ± 15.4			29.2 ± 15.5			44.8 ± 20.9			22.5 ± 18.0		
② Yes	22.7 ± 12.7			25.2 ± 12.8			39.3 ± 16.1			16.2 ± 11.6		
Use of lifting aids		2.55	0.004 **		2.15	0.003 **		2.73	0.172		2.60	0.002 **
① No	26.2 ± 15.4			28.7 ± 15.2			44.2 ± 19.3			20.8 ± 16.4		
② Yes	21.9 ± 10.5			24.3 ± 11.9			36.9 ± 15.6			15.7 ± 11.7		

Note: F: one-way ANOVA, *t*: independent t test, * *p* < *0*.05, ** *p* < 0.01, *** *p* < 0.001. The NMQ scores for sex, BMI, seniority, and working hours in the four areas did not reach statistically significant differences.

**Table 4:** Hierarchical analysis of factors affecting MSDs among nursing aides in LTCFs (*n* = 308).

Category	Variable	β	t	R^2^	ΔR^2^	Change in F Value
(Model 1)	Vietnamese vs. Taiwaness	0.254	4.178 ***	0.260	0.260	8.58 ***
	>65 y/o vs. 20–34 y/o	0.155	2.601 **			
	Two chronic diseases	0.309	5.569 ***			
	Middle vs. Northern	−0.157	−2.580 ***			
Job background variables (Model 2)	Vietnamese vs. Taiwaness	0.234	3.891 ***	0.352	0.092	5.04 ***
	>65 y/o vs. 20–34 y/o	0.170	2.780 **			
	Two chronic diseases	−0.266	4.908 ***			
	Middle vs. Northern	−0.155	−2.511 *			
	Rest time	−0.151	−2.365 *			
	Protective aids	−0.125	−2.328 *			
	Lifting aids	−0.191	−3.616 ***			
Work-related psychosocial factors (Model 3)	Vietnamese vs. Taiwaness	0.211	3.550 ***	0.421	0.070	6.73 ***
	>65 y/o vs. 20–34 y/o	0.125	2.050 *			
	Exercise ≥ 3 time/week vs. None	−0.099	−2.004 **			
	Two chronic diseases	0.185	3.423 **			
	Middle vs. Northern	−0.159	−2.593 *			
	Rest time	−0.136	−2.196 *			
	Lifting aids	−0.154	−3.029 **			
	Coworker support	−0.146	−2.079 *			
	Psychological job demands	0.164	2.798 **			

Note: hierarchical regression analysis, *t:* independent t test, R^2^: Coefficient of determination, F: one-way ANOVA * *p* < 0.05, ** *p* < 0.01, *** *p* < 0.001.

## Data Availability

Not applicable.
